# Prevalence and cost implications of broadening asthma biologic eligibility criteria^☆^

**DOI:** 10.1016/j.waojou.2026.101449

**Published:** 2026-08-19

**Authors:** Freda Yang, Benjamin P. Geisler, Grainne d’Ancona, Bohee Lee, David J. Jackson, Chloe I. Bloom

**Affiliations:** a National Heart and Lung Institute, Imperial College London, London, UK; b Guy's & Brompton Asthma Network, Royal Brompton Hospital, Guy's and St Thomas' NHS Foundation Trust Hospital, London, UK; c Department of Health Management and Health Economics, University of Oslo, Oslo, Norway; d Guy's & Brompton Asthma Network, Guy's Hospital, Guy's and St Thomas' NHS Foundation Trust Hospital, London, UK; e School of Immunology and Microbial Sciences, King's College London, London, UK

**Keywords:** Biologic, Severe asthma, Exacerbations, Anti-TSLP, Anti-IL5

## Abstract

**Rationale:**

Eligibility for biologic therapy in severe asthma varies internationally because government-funded schemes apply stricter criteria than pivotal trials. While relaxing eligibility thresholds may expand access, the effects on prevalence and healthcare costs remain uncertain.

**Objective:**

To quantify how alternative biologic eligibility criteria affect prevalence, clinical characteristics, and budget impact in adults with asthma.

**Methods:**

We studied adults with active asthma (2004–2021) using nationally representative primary care and hospital records. Biologic eligibility was assessed yearly using asthma severity (inhaled corticosteroid dose, additional controllers, rescue therapy) and exacerbations (oral corticosteroid prescriptions, emergency visits and hospitalisations). We assessed prevalence using United Kingdom (UK) criteria (severe asthma, ≥3 exacerbations), Global criteria (severe asthma, ≥1 or ≥2 exacerbations) and RCT criteria (moderate asthma, ≥1 or ≥2 exacerbations). Anti-IL-5/5Rα eligibility was evaluated using blood eosinophil counts. Budget impact was modelled over 5 years.

**Results:**

Among 1,306,307 asthma patients, 7.2% had severe asthma. Under UK criteria, 19,093 patients were biologic-eligible (20.4% of severe asthma; 1.5% of all asthma). Global criteria increased eligibility to 2.4% (≥2 exacerbations) to 4.3% (≥1 exacerbation), while RCT criteria expanded eligibility to 4.7% (≥2 exacerbations) and 9% (≥1 exacerbation). Among patients with eosinophil data, 38–45% met anti-IL-5/5Rα criteria. UK-eligible patients had higher prevalence of lower respiratory tract infections, pneumonia, and bronchiectasis. Five-year costs were £261 M under UK criteria, £436–767 M under Global criteria, and £878M-1.6B under RCT criteria.

**Conclusion:**

Only one-fifth of patients with severe asthma met UK biologic eligibility. Global criteria tripled eligibility and RCT criteria increased it six-fold, with substantial budget implications.

## Introduction

Asthma affects over 260 million people worldwide and remain inadequately controlled in many despite treatment with inhaled corticosteroids (ICS) and long-acting β2-agonists (LABA).^1^^,^^2^ In these patients, biologic therapies targeting type-2 (T2) inflammation can reduce exacerbation frequency and improve quality of life.^3^ Six biologics are approved for asthma, but patient access is constrained by national prescribing criteria more stringent than those used in randomised controlled trials (RCTs).^4^^,^^5^

Asthma biologic RCTs typically enrol patients with at least 1 prior-year exacerbation while receiving moderate-to-high-dose ICS plus an additional controller, whereas many national guidelines require high-dose ICS, additional controllers, and more frequent exacerbations.^3^^,^^4^ In the United Kingdom, NICE stipulates ≥3 exacerbations in 12 months while receiving high-dose ICS plus additional controllers—higher than most countries, where ≥1 or ≥2 exacerbations typically suffice.^4^^,^^6^, ^7^, ^8^, ^9^, ^10^

Higher exacerbation thresholds have been recommended based on cost-effectiveness, and higher biomarker thresholds (blood eosinophils ≥300 cells/μL for anti-IL-5/5Rα therapies) enrich for likely responders.^11^, ^12^, ^13^ However, stricter thresholds may defer biologic initiation, exposing patients to repeated exacerbations, cumulative systemic corticosteroid exposure, and potential "too late" asthma with irreversible airway remodelling.^5^^,^^6^ This highlights the challenge of balancing cost-effectiveness and clinical rationale for earlier intervention.

The first omalizumab biosimilar was approved by the European Medicines Agency in 2024 and the United States (US) Food and Drug Administration (FDA) in 2025.^14^, ^15^, ^16^ As patents expire and more biosimilars become available, falling prices may shift eligibility thresholds and expand access. Quantifying how alternative definitions change the size and profile of eligible patients, and the associated costs, is a pressing policy question that no prior study has addressed using nationwide linked data.

Using nationwide UK primary care and hospital data, we estimate biologic eligibility prevalence under current UK clinical guidelines criteria, 2 international comparators, and 2 RCT-based benchmarks. We examine how thresholds affect who qualifies and estimate the budget impact for the National Health Service.

## Methods

### Data source and study population

We used the Clinical Practice Research Datalink (CPRD), one of the world's largest primary care databases, with patient-level linkage to Hospital Episode Statistics (HES) for emergency department attendances and hospital admissions. CPRD includes anonymised data on demographics, prescriptions, and comorbidities from general practices across the UK, covering approximately 20% of the national population.

Patients from 1471 general practices meeting CPRD quality standards and contributing data between January 1, 2004 and March 31, 2021 were included. Adults aged ≥18 years with asthma were identified using validated diagnostic codes.^17^ To ensure active disease, inclusion required at least 1 asthma prescription. Study entry was the latest of the asthma diagnosis date, the first asthma prescription, or the eighteenth birthday. Follow-up ended at the earliest of transfer out of a CPRD practice, the last asthma prescription, or death. Patients with <365 days of follow-up were excluded to allow at least 1 full year of observation. Patients with comorbid chronic obstructive pulmonary disease were excluded.

### Asthma medications, severity and exacerbations

Asthma controller medications included ICS, LABA, leukotriene receptor antagonists (LTRA), and theophylline. ICS dose was expressed as beclometasone dipropionate (BDP) equivalents and categorised as low (200–500 μg/day), medium (600–800 μg/day), or high (1000–2000 μg/day) according to 2024 NICE/British Thoracic Society guidance and Global Initiative for Asthma (GINA).^18^^,^^19^ Severe asthma was defined as requiring any form of rescue treatment (SABA and systemic corticosteroids) despite high-dose ICS plus 1 other controller. Moderate asthma was defined as requiring medium-dose ICS (Supplement Table E1). Adherence was considered adequate if patients obtained ≥70% of expected annual prescriptions (≥9 inhalers per year) in line with National Health Service (NHS) England requirements before biologic initiation.^20^

Exacerbations were defined as any of a short course of oral corticosteroid prescription, emergency department attendance or hospital admission for asthma. Events occurring within 14 days were considered a single episode.

### Definitions for biologic eligibility

Follow-up was divided into consecutive 12-month intervals starting from the study entry date. We excluded terminal intervals shorter than 12 months and patients with <12 months of total follow-up to allow for adequate seasonal coverage. For each interval, we determined the asthma severity and total exacerbations, then assessed biologic eligibility under 5 prespecified scenarios, UK clinical guidelines (NICE), Global1, Global2, RCT1, and RCT2, defined as:

• UK: severe asthma and ≥3 exacerbations.
• Global1: severe asthma and ≥1 exacerbation.
• Global2: severe asthma and ≥2 exacerbations.
• RCT1: moderate asthma and ≥1 exacerbation.
• RCT2: moderate asthma and ≥2 exacerbations.

The UK scenario reflects the minimum NHS requirements for asthma biologics.^6^ The Global scenarios derive from a systematic review of global prevalence in biologic eligibility^10^ and the RCT scenarios from a systematic review of trial inclusion criteria.^3^ Full specifications and rationale are in Supplementary Tables E2-E4.

The first 12-month interval in which a patient met biologic criteria was defined as "eligible year". Subsequent intervals were censored because in clinical practice, patients would initiate a biologic, switch agents, or discontinue if ineligible for alternatives, so eligibility is counted once.

Of the 3 biomarkers commonly used for eligibility (total IgE, blood eosinophils, and FeNO), only blood eosinophils were available. We applied thresholds of 400 and 300 cells/μL (anti-IL-5/5Rα therapies) and 150 cells/μL (anti-IL-4/4Rα therapies). For each patient, we extracted (i) the highest blood eosinophil count across follow-up and (ii) the highest eosinophil count during eligible year.

### Covariates

Covariates were identified using validated Read codes (available on https://github.com/BoheeLEE/SevereAsthma). Smoking history was classified as ever-smoker (current and former) and ex-smokers. Body mass index (BMI) was taken as the value closest to, and prior to, the biologic eligibility date. Asthma-related comorbidities included atopic dermatitis, rhinitis, hay fever, aspirin-exacerbated respiratory disease. Lower respiratory tract infection, pneumonia, and bronchiectasis, anxiety, depression, and cardiovascular disease (ischaemic heart disease, hypertension, and heart failure) were also identified from general practice records.

### Statistical analyses

Analyses were conducted in R (v4.3.2). Baseline characteristics are summarised as means (standard deviation, SD) or medians (interquartile ranges, IQR) for continuous variables, and counts (percentage) for categorical variables. Biologic eligibility prevalence was calculated as (i) the proportion of all patients with active asthma meeting each definition and (ii) the proportion of severe asthma patients meeting UK, Global1, and Global2 definitions.

Between-group differences were assessed using logistic regression model fitted with generalised estimating equations (GEE) to account for repeated observations from patients appearing in multiple groups. We report odds ratios (ORs) and 95% confidence intervals (CIs) for each cohort, using the UK group as the reference.

### Cost analyses

Biologic costs vary by administration route (subcutaneous or intravenous), setting (clinic or home), and dosing frequency (every 2–8 weeks). List prices were obtained from the British National Formulary and NICE committee documents (Supplement Table E5). Actual NHS acquisition prices are lower but confidential; we estimated costs using 75% discount to list prices, an illustrative net-price assumption reflecting that NHS procurement discounts can be substantial under confidential commercial arrangements.

Exacerbation costs were calculated according to setting: (i) primary care, (ii) ED, and (iii) hospital admission. The primary-care unit cost comprised 1 general practitioner consultation plus a prednisolone pack (40 mg daily for 5 days). Emergency department (ED) unit costs came from the King's Fund 2023/2024 estimates,^21^ and hospital admission costs from the 2020/21 NHS National Cost Collection for non-elective admissions, as cited in NICE TA880 (Supplement Table E6).

For each patient, we calculated the mean exacerbation treatment cost during the eligible year. After biologic initiation, the net annual per-patient cost equalled the biologic drug cost plus remaining exacerbation costs, assuming a 75% reduction in exacerbations.^22^, ^23^, ^24^, ^25^

Current biologic treatment penetration, defined as the proportion of eligible patients receiving biologic therapy, was estimated at 16% in 2023.^26^ For each of the 5 eligibility criteria, we assumed a baseline treatments penetration of 20% and modelled cumulative costs over a 5-year horizon. Outcomes included:^1^ annual biologic treatment costs,^2^ post-biologic exacerbation costs,^3^ budget impact to National Health Service (NHS) England accounting for exacerbations cost-savings following biologic initiation, and^4^ per-member-per-month costs, which represents the additional costs per person in England.

## Results

### Eligibility under UK, global, and RCT criteria

Among 1,306,307 adults with active asthma (median follow-up 6 years; IQR 3–12), the median age at study entry was 38 years (IQR 26–53) and 57.9% (n = 756,733) were female. 7.2% (n = 93,652) met criteria for severe asthma.

**Fig. 1:**
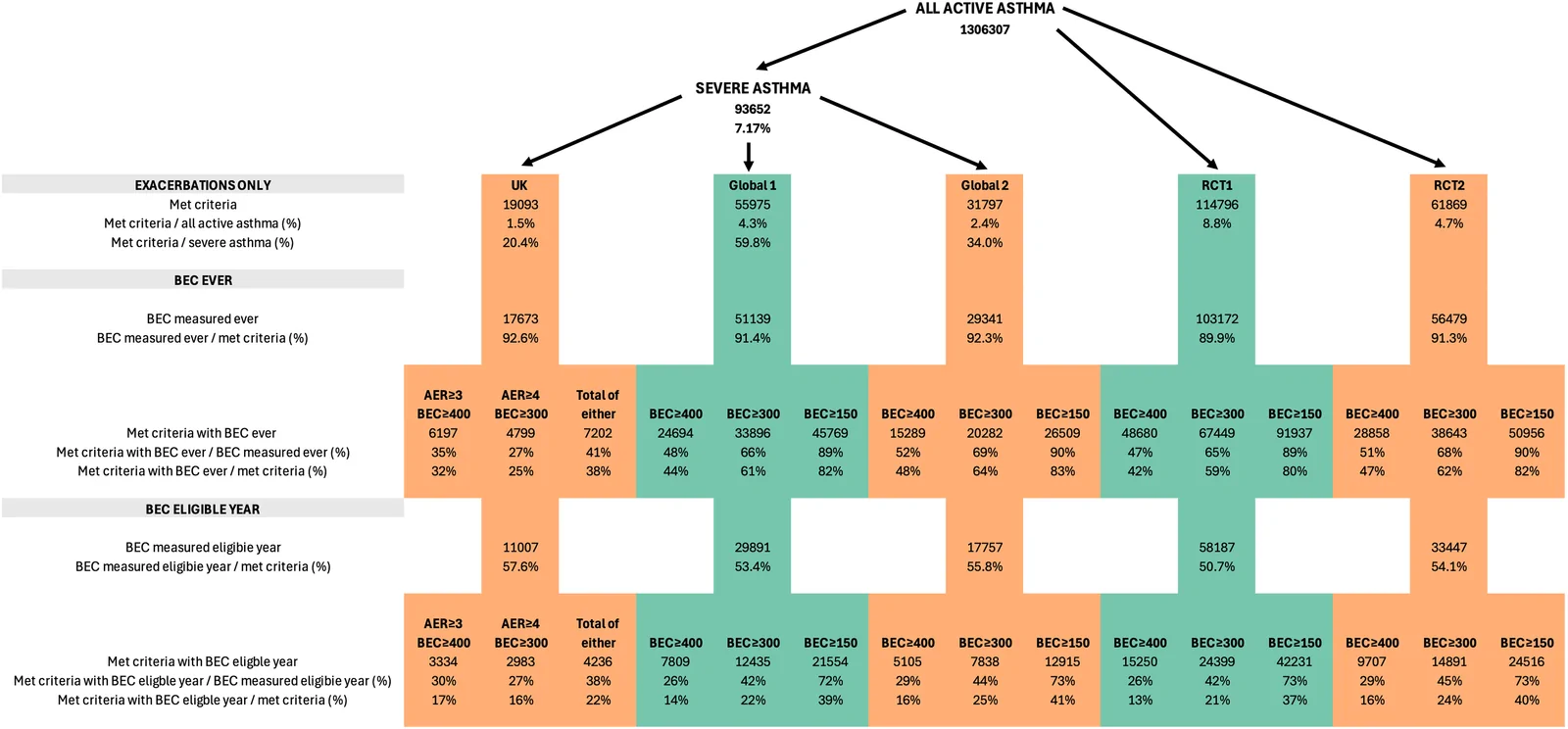
Prevalence of biologic eligible patients according to various criteria (UK, Global 1, Global 2, RCT 1 and RCT 2) and proportion of patients eligible for anti-IL5/5Ra treatments according to highest ever recorded blood eosinophil count and during biologic eligible year (12 months)

**Fig. 2:**
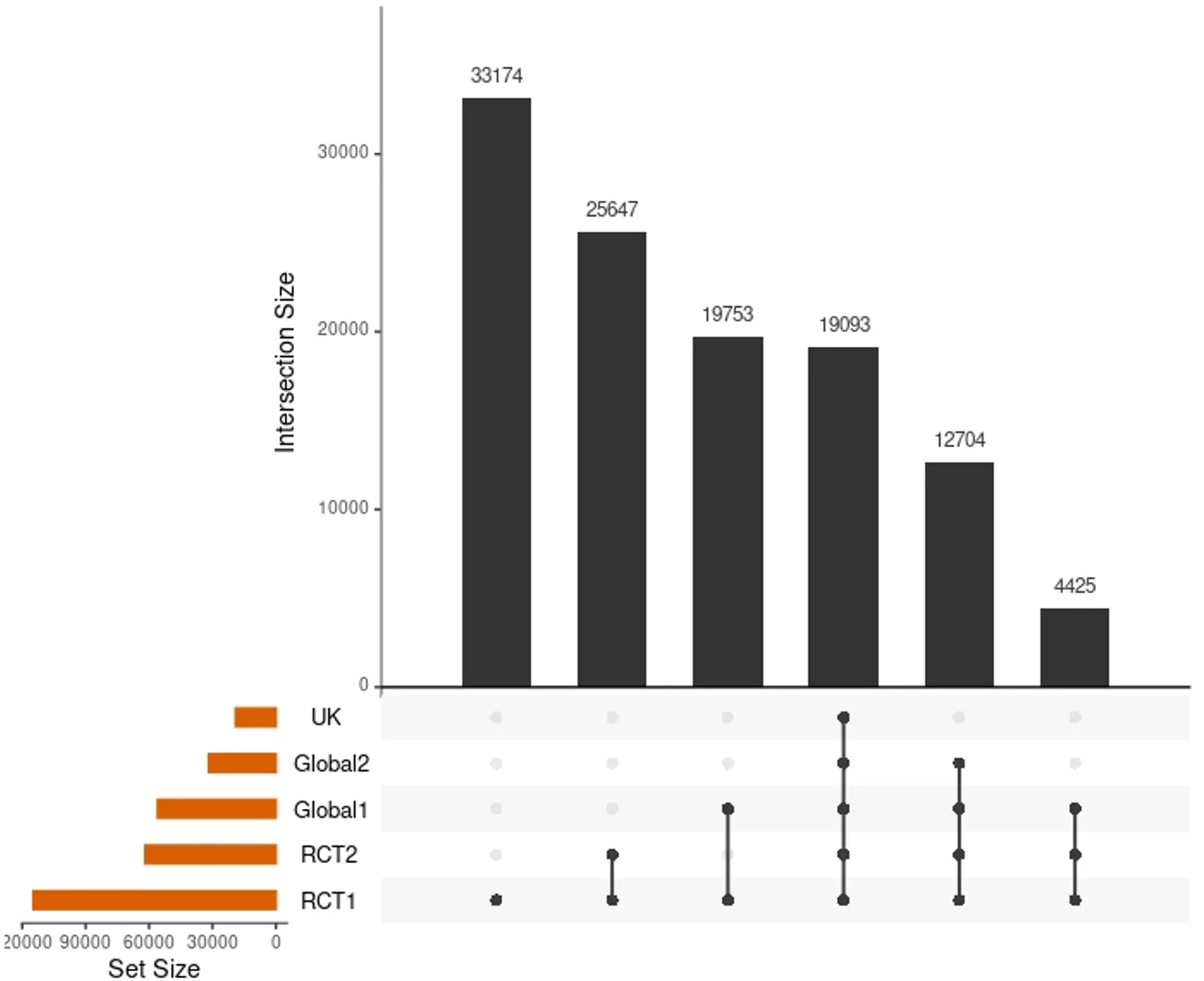
UpSet plot illustrating patient overlap across eligible cohorts defined by different eligibility criteria. Vertical black bars (top) represent the number of patients that are either unique to the eligibility group (black dot with no connected line) or patients that overlap multiple eligibility groups (joined black dots for each group they are in). Horizontal orange bars (left) indicate the total patient population size (set size) for each group (UK, Global2, Global1, RCT2, and RCT1)

Representing 20.4% of the severe asthma population and 1.5% of all adults with asthma, 19,093 patients met the minimum UK eligibility criteria for biologic therapy. The number eligible increased to 31,797 (Global2) and 55,975 (Global1) when exacerbation thresholds were relaxed to ≥2 (Global2) and ≥1 (Global1) in 12 months. This corresponds to 34.0% (Global2) and 59.8% (Global1) of those with severe asthma and 2.4% (Global2) and 4.3% (Global1) of all adults with asthma. RCT-based definitions, which include moderate asthma, expanded eligibility to 61,869 (RCT2) and 114,796 (RCT1) individuals, which corresponds to 4.7% and 8.8% of all adults with asthma, respectively (Fig. 1). Overlap between these 5 criteria is shown in Fig. 2.

### Blood eosinophils and anti-IL5/5Ra eligibility

Blood eosinophil data were available for 68.2% (n = 891,489) of all patients at any time during follow-up. Among those meeting UK biologic criteria, 92.6% had a historical eosinophil count recorded at any time, but only 57.6% had a count measured during the eligible year. The proportions with any historical count and with a count during the eligible year were similar among those meeting Global1, Global2, RCT1, and RCT2 criteria (Fig. 1).

Among patients with blood eosinophils available during the eligible year, 38% of those qualifying for biologics in the United Kingdom were eligible for anti-IL5/5Rα therapies. Using a threshold of ≥300 cells/μL during the eligible year, 42% (Global1), 44% (Global2), 42% (RCT1), and 45% (RCT2) of biologic-eligible patients with available blood tests qualified for anti-IL5/5Rα therapies. These proportions increased up to 69% when considering all historical eosinophil counts (Fig. 1). Overlap across groups is shown in Supplement Figure E1. Results for alternative thresholds (≥150 and ≥ 400 cells/μL) are shown in Fig. 1.

### Clinical characteristics according to UK, global, and RCT criteria

Median age (approximately 56 years) and the proportion of females (around 69%) were similar across the UK, Global1, Global2, RCT1, and RCT2 cohorts. Smoking history and BMI were likewise comparable. Comorbidity profiles, including atopic dermatitis, rhinitis, hay fever, anxiety, and cardiovascular disease, were broadly similar across all 5 groups. However, UK-eligible patients had a higher prevalence of lower respiratory tract infection, pneumonia and bronchiectasis than the Global1, Global2, RCT1 and RCT2 cohorts (Table 1, Supplement Figure E2).

**Table 1:** Demographic and clinical characteristics of biologic eligible cohorts according to UK, global and RCT criteria.

	UK N = 19,093	Global1 N = 55,975	Global2 N = 31,797	RCT1 N = 114,796	RCT2 N = 61,869
Age at meeting biologic criteria (years)	56.6 (43.5, 70.2)	56.5 (43.5, 69.8)	56.6 (43.5, 70.4)	55.5 (42.5, 68.5)	56.0 (42.8, 69.5)
Female	13,445 (70%)	37,787 (68%)	22,098 (69%)	76,849 (67%)	42,869 (69%)
Ever smoker	13,175 (69%)	39,078 (70%)	22,086 (69%)	79,053 (69%)	42,406 (69%)
Ex-smoker	10,043 (53%)	29,596 (53%)	16,784 (53%)	60,221 (52%)	32,419 (52%)
Body mass index^a^	29.2 (25.1, 34.4)	29.0 (25.0, 34.1)	29.1 (25.1, 34.4)	28.7 (24.9, 33.6)	28.9 (25.0, 34.0)
Atopic dermatitis	5647 (30%)	15,860 (28%)	9262 (29%)	32,652 (28%)	18,103 (29%)
Rhinitis	3957 (21%)	10,849 (19%)	6392 (20%)	21,994 (19%)	12,276 (20%)
Hay fever	4441 (23%)	12,533 (22%)	7301 (23%)	25,492 (22%)	14,084 (23%)
AERD	27 (0.1%)	63 (0.1%)	37 (0.1%)	110 (<0.1%)	63 (0.1%)
LRTI	16,326 (86%)	45,378 (81%)	26,765 (84%)	89,745 (78%)	50,955 (82%)
Pneumonia	3067 (16%)	6979 (12%)	4595 (14%)	12,555 (11%)	7977 (13%)
Bronchiectasis	2302 (12%)	4332 (7.7%)	3158 (9.9%)	7120 (6.2%)	5096 (8.2%)
Anxiety	5933 (31%)	17,028 (30%)	9895 (31%)	33,691 (29%)	18,816 (30%)
Cardiovascular disease	9765 (51%)	28,715 (51%)	16,365 (51%)	56,057 (49%)	30,747 (50%)
Received a PAAP	16,741 (88%)	49,328 (88%)	27,944 (88%)	101,033 (88%)	54,417 (88%)

Results are shown as n (%) or median (Q1, Q3).

**Abbreviations:** AERD, aspirin-exacerbated respiratory disease; LRTI, lower respiratory tract infection; PAAP, personalised asthma action plan.

a Body mass index was taken from the most recent measurement before the end of the biologic-eligible year and was available for 94% of patients across the cohorts

### Projected biologic-associated costs

Among UK-eligible patients, 94% of exacerbations were managed in primary care, 5% led to hospital admission, and 1% were managed solely in the ED. Despite their relative low frequency, hospital admissions accounted for 80% of total exacerbation costs (primary care 17%, ED 3%). The average pre-biologic exacerbation cost per patient is £1037.

**Fig. 3:**
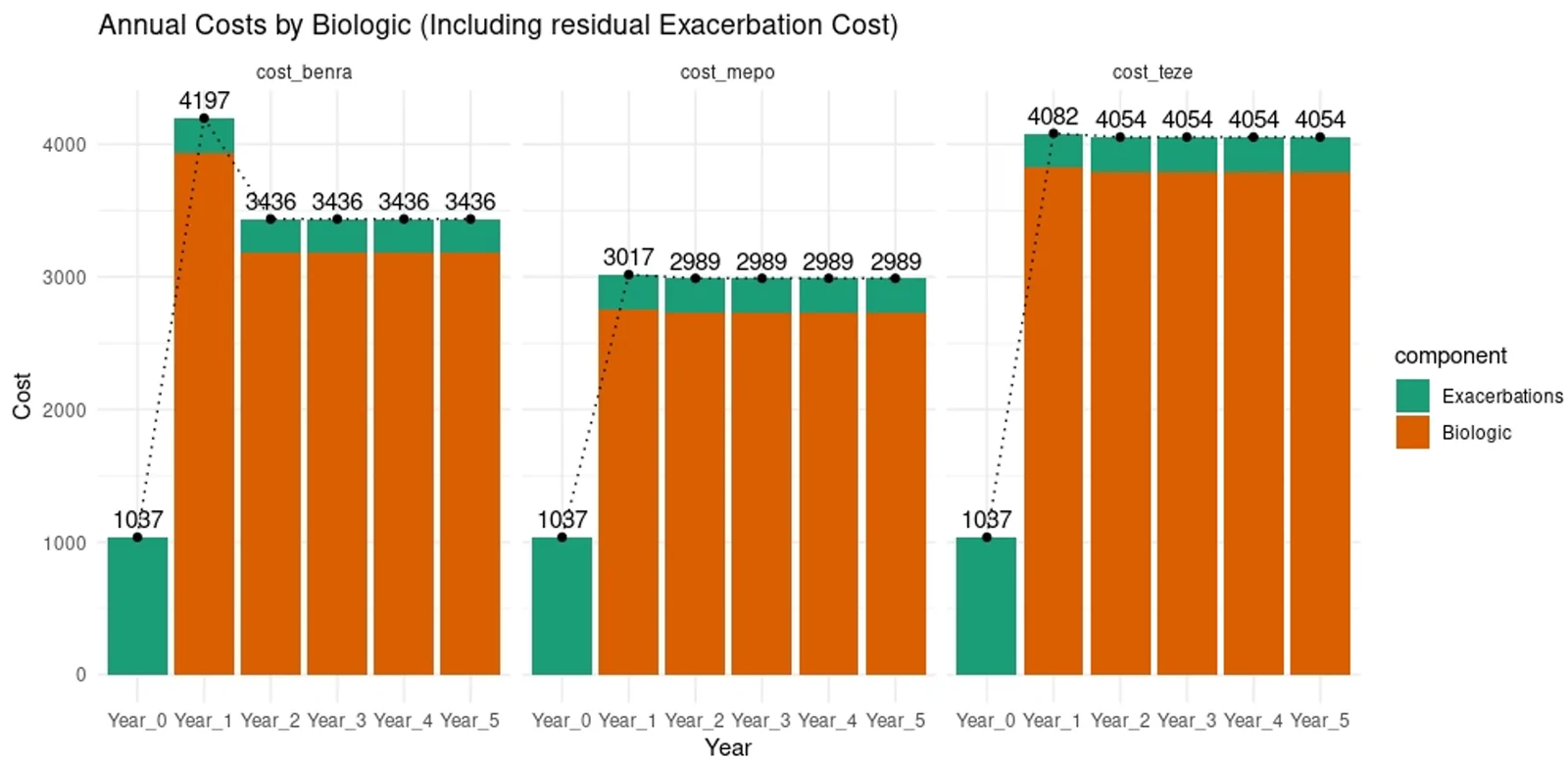
Projected per-patient costs by biologic over 5 years. *Year 0* reflects the 12 months before biologic initiation (eligible year). *Years 1–5* reflect time on biologic therapy. For each on-biologic year, total cost equals biologic cost (list price with a 75% discount) plus exacerbation related healthcare costs (assumed to be 75% lower than in *Year 0*)

Per patient, the total cost of tezepelumab and remaining post-treatment exacerbations was £4086 in the first year. The cost remained high in subsequent years (£4059 annually). Despite reductions in exacerbation costs, this exceeded the pre-biologic annual exacerbation cost of £1037. For benralizumab, the total first-year cost was £4,197, decreasing to £3436 annually in subsequent years. Mepolizumab had the lowest costs, with £3017 in Year 1 and £2989 annually thereafter. Over 5 years, cumulative per-patient costs (treatment plus residual exacerbations) were £20,298 for tezepelumab, £17,941 for benralizumab, and £14,973 for mepolizumab (Fig. 3).

**Fig. 4:**
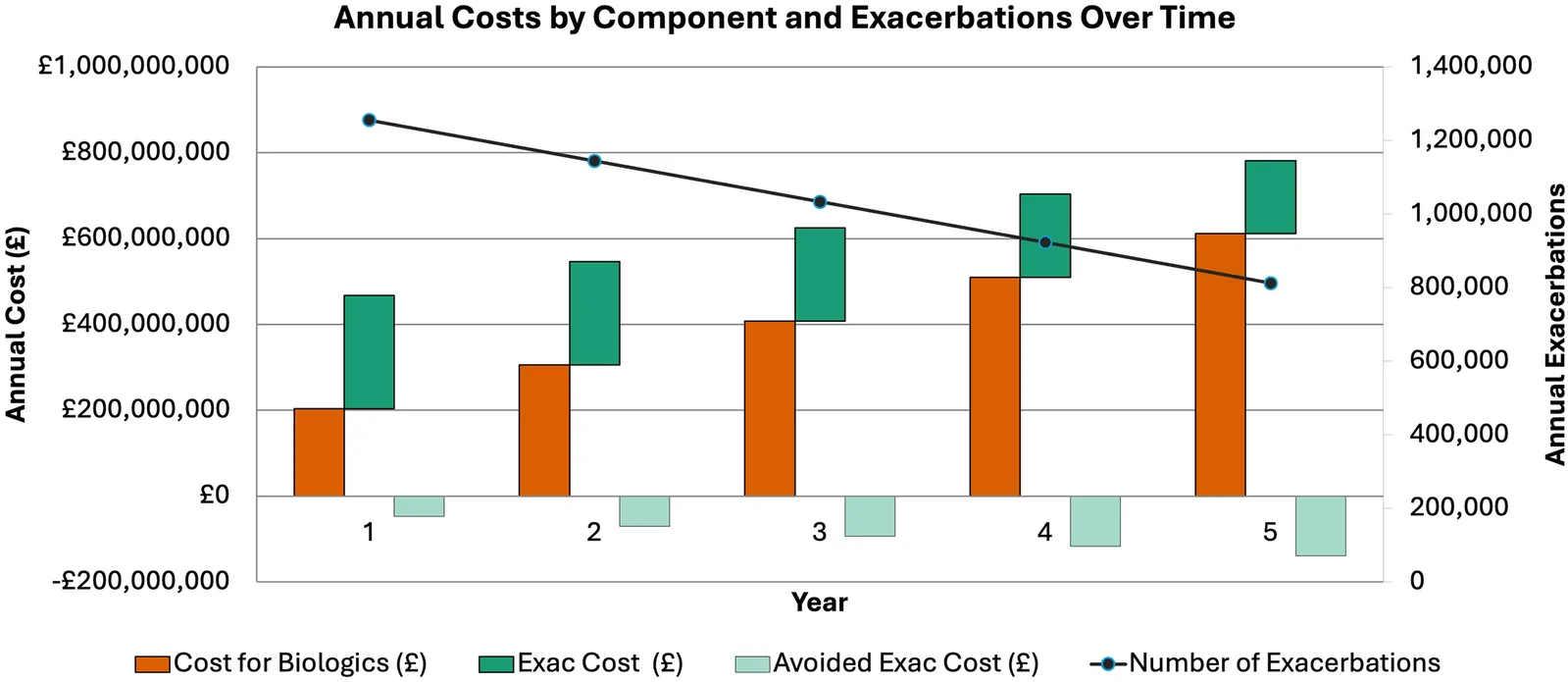
Annual costs of biologic treatment (orange bars), of post-treatment exacerbation costs (green bars), and of avoided exacerbation costs (white bars), as well as number of post-treatment exacerbations (line graph on the second y-axis) over a 5-year horizon for eligible patients in England meeting current UK biologic criteria (severe asthma with ≥3 exacerbations), starting from 20% biologic treatment penetration with 10% annual incremental increases, and corresponding annual cost-savings from avoided exacerbations

The estimated number of patients eligible for biologic treatment, projected to the entire England population, were 54,577 (UK criteria), 90,892 (Global ≥2 exacerbations), 160,005 (Global ≥1 exacerbation), 176,853 (RCT ≥2 exacerbations), and 328,144 (RCT ≥1 exacerbation). Assuming current biologic treatment penetration of 20% with 10% annual increments reaching 60% treatment penetration at 5 years, the total cost for biologic treatment over this period would be £339 M under current UK criteria, incorporating a 75% national discount (Fig. 4).

Using current UK biologic eligibility criteria, the budget impact of biologics (assuming a market share of 35%, 45%, and 20% for benralizumab, mepolizumab, and tezepelumab, respectively) to NHS England including the costs for biologics and the cost savings from avoided exacerbations was £261 M. This figure includes cumulative 5-year cost-savings from avoided exacerbations compared to no biologic treatment of £77 M (Fig. 4). Budget impact increased from £261 M to £436 M (Global2), £767 M (Global1), £878 M (RCT2), or £1.573B (RCT1) if criteria were broadened (Supplement Table E7, Figure E3). Corresponding per-member-per-month expenses ranged from 7 to 45 pence (Supplement Figure E4).

## Discussion

In this nationwide UK cohort, only 1.5% of adults with asthma were eligible for biologics based on current national criteria. This increases to 2.4–4.3% with international definitions and up to 9% with RCT-based criteria. Among those with severe asthma, only 1 in 5 qualifies for biologic therapy under current UK criteria. Lowering exacerbation thresholds would substantially expand access but increase costs: from £261M under current criteria to £767M–1.6B with broader definitions over 5 years. Blood eosinophils were recorded in around two-thirds of biologic-eligible patients during the eligible year. Fewer than half of the patients who qualify based exacerbation criteria met threshold for anti-IL-5/5Rα therapies. These findings provide practical benchmarks for policymakers as biologic pricing evolves. Earlier eligibility will likely depend on lower drug prices and tighter biomarker strategies to concentrate treatment where benefit is greatest.

This is the first nationwide estimate of biologic eligibility using linked primary and secondary care data. Prior reports largely come from single specialist centres or cohorts enriched for biologic candidates.^27^, ^28^, ^29^ Published estimates range from 2 to 12%, with 2% reported in an adult-onset Finnish cohort,^30^ 3% in a large US population,^31^ and 12% in an Italian/German cohort^32^ Our 2.4–4.3% under global criteria sits at the conservative end, likely reflecting our use of routine primary-care data which mitigates referral bias.

Among ATS/ERS-defined severe asthma patients, biologic eligibility estimates vary from 24% in a large European study^33^ to >90% in tertiary-centre cohorts.^34^, ^35^, ^36^ In our study, only 20.4% met the ≥3 exacerbation threshold, increasing to 34.0% and 59.8% when threshold were lowered to ≥2 and ≥ 1 exacerbations respectively. A difference of just 1 exacerbation annually accounts for much of this variation. We are confident that these estimates are accurate as our finding of 7.2% severe asthma prevalence aligns with previous reports, supporting generalisability.^37^, ^38^, ^39^

Most biologic-eligible patients had a historical eosinophil count, but counts were often not measured during the eligible year, preventing qualification for anti-IL-5/5Rα therapy. Guidelines during the study period (2004–2021) did not require primary care to check eosinophils; full blood counts were likely ordered to exclude alternative diagnoses. Although eosinophil testing is now recommended, uptake in routine practice remains uncertain.^18^^,^^19^

Broadening eligibility from current UK criteria to international or RCT-based thresholds identified populations with a lower prevalence of lower respiratory tract infection, pneumonia and bronchiectasis. Chronic uncontrolled asthma increases bronchiectasis risk^40^ and co-existing bronchiectasis attenuates biologic responses.^41^^,^^42^ These findings align with the “too-late” asthma concept, wherein delayed treatment reduces the likelihood of remission and this maybe related to non-T2 airway inflammation associated with chronic asthma.^43^^,^^44^ Earlier eligibility may protect against bronchiectasis progression and preserve biologic responsiveness, but requires prospective evaluation against health-service costs.

We undertook pragmatic cost comparisons between standard care and biologic therapy, accounting for expected reductions in asthma exacerbations. Even assuming a 75% reduction in exacerbations annually, total management costs with biologics remained substantively above standard care. Although NHS acquisition prices are confidential, NICE appraisals indicate asthma biologics exceed cost-effectiveness thresholds, and US ICER reviews reached similar conclusions even under the favourable assumptions.^45^ Our estimates exclude QALYs gained and potential offsets from reduced steroid-related comorbidities, rescue inhaler use, and severe asthma service costs.

Spontaneous remission in severe asthma is uncommon,^46^ making biologics long-term and potentially lifelong for many. We estimated £261M over 5 years under current UK criteria, rising to nearly £1B with global criteria or £1.6B with RCT criteria. These costs extend well beyond 5 years given the median biologic initiation age of 50.^47^ Two potential solutions exist: reduce biologic costs through biosimilar uptake, or implement tighter biomarker-based targeting to maximise response and remission. Although imperfect, routine use of blood eosinophils and FeNO in pre-biologic workup can enrich for treatment responders.^48^

Study strengths include nationwide scale, objective identification from linked prescribing and hospital data (avoiding recall bias), and granular characterisation of treatment intensity aligned with UK practice. We removed duplicate OCS prescriptions within 14 days to minimise exacerbation misclassification.

Limitations include difficulty distinguishing daily OCS use from frequent bursts, though such patients typically accrue ≥12 prescriptions annually and would qualify for biologics regardless. Some OCS prescriptions may reflect alternative diagnoses, such as rheumatoid arthritis. For chronic rhinosinusitis, another potential indication, this is rarely prescribed in primary care. "Rescue packs" of OCS could lead to prescriptions preceding actual exacerbations, though our study design meant these events were counted without duplication, just with a slight delay. The absence of IgE and FeNO data restricted assessment of omalizumab and dupilumab eligibility. Consequently, we were unable to estimate the proportion of patients who may be eligible for these biologics, which introduces uncertainty into the overall cost projections. Lack of specialist confirmation and lung function data may overestimate severe asthma prevalence, though we partially addressed this by excluding COPD.

In conclusion, only a small minority of adults with asthma currently meet UK biologic criteria. Relaxing exacerbation thresholds would markedly increase access but also spending. Routine blood eosinophil measurements should be embedded in primary care to enrich for biologic responders, particularly if exacerbation criteria were broadened. Given the substantial budget impact, priority should be to identify patients at highest risk of persistent frequent exacerbations. The optimal timing for biologic initiation and strategies for de-escalation or discontinuation require further evaluation.

## Confirmation of Unpublished work

This manuscript is original, has not been published previously, and is not under consideration for publication elsewhere.

## Guarantor statement

Freda Yang is the guarantor of this study and takes full responsibility for the integrity of the data and the accuracy of the analyses.

## Notation of prior abstact publication/presentation

This work was previously presented at the European Respiratory Society International Congress 2025, held in Amsterdam, under the title: Impact of national clinical guidelines versus RCT criteria on biologic eligibility in asthma. European Respiratory Journal 2025; 66 (suppl 69): PA1425.

## Ethics Statement

CRPD has generic ethnical approval from the Health Research Authority Research Ethics Committee (East Midlands – Derby, reference number 05/MRE04/87). Data use was approved by Independent Scientific Advisory committee (ISAC) for Medicines and Healthcare products Regulatory Agency (MHRA) (protocol 23_002634). Data management was provided by the Big Data and Analytical Unit (BDAU) at the Research Computing Services within Imperial.

## Contributions

Acquisition of data: FY, CIB.

Analysis of data: FY, BPG.

Drafting of the manuscript: FY.

Interpretation of results and critical revision of the manuscript: FY, CIB, GA, BL, DJJ, BPG.

Obtained funding: FY, CIB.

Supervision: CIB.

## Disclosure of the use of Generative AI and AI-assisted Technologies

ChatGPT (OpenAI) was used only to improve clarity and readability of the text. It did not generate scientific content, clinical advice, or any figures, images, or artwork.

## Funding

This research was funded by the 10.13039/501100000272National Institute for Health Research (10.13039/501100000272NIHR) 10.13039/501100013342Imperial Biomedical Research Centre (BRC). FY is supported by an 10.13039/501100000272NIHR Clinical Lectureship and acknowledges infrastructure support for this research from the 10.13039/501100013342NIHR Imperial BRC.

## Conflicts of interest

**FY** has received honoraria for attending meetings and speaker fees from AstraZeneca and GlaxoSmithKline; is a member of the British Thoracic Society asthma specialist advisory group. **BPG** reports no relevant conflicts of interest. **GA** reports no relevant conflicts of interest. **BL** reports support for the present study and grants from Asthma + Lung UK. **DJJ** has received advisory board and speakers fees from AstraZeneca, Boehringer Ingelheim, Novartis, Teva, GSK, Sanofi-Regeneron, and Chiesi. **CIB** has received research funding from the NIHR, Medical Research Council, European Respiratory Society, Asthma + Lung UK and AstraZeneca, outside the scope of this work; leadership roles in the British Thoracic Society and UK Primary Care Respiratory Society.
