# Supplementary material for: Prevalence and cost implications of broadening asthma biologic eligibility criteria
Source: World Allergy Organ J. 2026 Aug 19;19(9):101449. doi: 10.1016/j.waojou.2026.101449 (PMC13520886; doi:10.1016/j.waojou.2026.101449)
Supplement: Supplementary file 1 [file mmc1.docx]

**SUPPLEMENTARY DOCUMENT (FIGURES)**

**Figure E1.** UpSet plot illustrating patient overlap across eligible cohorts defined by different eligibility criteria for anti-IL-5/5Ra. Vertical black bars (top) represent the number of patients that are either unique to the eligibility group (black dot with no connected line) or patients that overlap multiple eligibility groups (joined black dots for each group they are in). Horizontal orange bars (left) indicate the total patient population size (set size) for each group (UK anti-IL-5/5Ra criteria, Global2, Global1, RCT2, and RCT1 using the ≥300 cells/µL blood eosinophil count threshold.

**Figure E2.** Bar plots showed prevalence of LRTI, pneumonia, bronchiectasis in each cohort (UK, Global1, Global2, RCT1, RCT2), with Forest plots showed odds ratios (ORs) for each comorbidity from population-averaged logistic regression using generalised estimating equations (GEE), with the cohort meeting UK eligibility criteria as the reference group. Error bars indicate 95% CIs.

**Figure E3.** Annual costs of biologic treatment and post-treatment exacerbations over a 5-year horizon for all asthma patients in England meeting **(A-B)** Global and **(C-D)** RCT criteria, starting from 20% biologic treatment penetration with 10% annual incremental increases, and corresponding annual cost-savings from avoided exacerbations.

(A) **Global 2 (n=90,892)**

Annual Costs by Component and Exacerbations Over Time

Annual Cost (£)

Annual Exacerbations

| Year | Cost for Biologics (£) | Exac Cost (£) | Avoided Exac Cost (£) |
| --- | --- | --- | --- |
| 1 | 203780705 | 263456153 | -46492262 |
| 2 | 305671087 | 240210022 | -69738393 |
| 3 | 407561440 | 216963891 | -92984525 |
| 4 | 509451792 | 193717760 | -116230656 |
| 5 | 611342145 | 170471628 | -139476787 |

|  | Number of Exacerbations |
| --- | --- |
| 1 | 1255151 |
| 2 | 1144402 |
| 3 | 1033654 |
| 4 | 922905 |
| 5 | 812156 |

(B) **Global1 (n=160,005)**

Annual Costs by Component and Exacerbations Over Time

Annual Cost (£)

Annual Exacerbations

| Year | Cost for Biologics (£) | Exac Cost (£) | Avoided Exac Cost (£) |
| --- | --- | --- | --- |
| 1 | 203780705 | 263456153 | -46492262 |
| 2 | 305671087 | 240210022 | -69738393 |
| 3 | 407561440 | 216963891 | -92984525 |
| 4 | 509451792 | 193717760 | -116230656 |
| 5 | 611342145 | 170471628 | -139476787 |

|  | Number of Exacerbations |
| --- | --- |
| 1 | 1255151 |
| 2 | 1144402 |
| 3 | 1033654 |
| 4 | 922905 |
| 5 | 812156 |

(C) **RCT2 (n=176,853)**

Annual Costs by Component and Exacerbations Over Time

Annual Cost (£)

Annual Exacerbations

| Year | Cost for Biologics (£) | Exac Cost (£) | Avoided Exac Cost (£) |
| --- | --- | --- | --- |
| 1 | 203780705 | 263456153 | -46492262 |
| 2 | 305671087 | 240210022 | -69738393 |
| 3 | 407561440 | 216963891 | -92984525 |
| 4 | 509451792 | 193717760 | -116230656 |
| 5 | 611342145 | 170471628 | -139476787 |

|  | Number of Exacerbations |
| --- | --- |
| 1 | 1255151 |
| 2 | 1144402 |
| 3 | 1033654 |
| 4 | 922905 |
| 5 | 812156 |

(D) **RCT1 (n=328,144)**

Annual Costs by Component and Exacerbations Over Time

Annual Cost (£)

Annual Exacerbations

| Year | Cost for Biologics (£) | Exac Cost (£) | Avoided Exac Cost (£) |
| --- | --- | --- | --- |
| 1 | 203780705 | 263456153 | -46492262 |
| 2 | 305671087 | 240210022 | -69738393 |
| 3 | 407561440 | 216963891 | -92984525 |
| 4 | 509451792 | 193717760 | -116230656 |
| 5 | 611342145 | 170471628 | -139476787 |

|  | Number of Exacerbations |
| --- | --- |
| 1 | 1255151 |
| 2 | 1144402 |
| 3 | 1033654 |
| 4 | 922905 |
| 5 | 812156 |

**Figure E4.** Per-member-month analysis of the budget impact of biologic treatments net costs, including cost savings from avoided exacerbations.
