# Supplementary material for: Prevalence and cost implications of broadening asthma biologic eligibility criteria
Source: World Allergy Organ J. 2026 Aug 19;19(9):101449. doi: 10.1016/j.waojou.2026.101449 (PMC13520886; doi:10.1016/j.waojou.2026.101449)
Supplement: Supplementary file 2 [file mmc2.docx]

**SUPPLEMENTARY DOCUMENT (TABLES)**

**Table E1.** Equivalent dose categories for inhaled corticosteroids.

|  | **High dose** | **Medium dose** | **Low dose** |
| --- | --- | --- | --- |
| **Inhaled corticosteroids** | | | |
| Beclometasone DP | 1000-2000mcg/day | 600-800mcg/day | 200-500mcg/day |
| Beclometasone DP (Fostair) | 500-800mcg/day | 300-400mcg/day | 100-200mcg/day |
| Budesonide | 1000-1600mcg/day | 600-800mcg/day | 200-400mcg/day |
| Ciclesonide | 400-640mcg/day | 240-320mcg/day | 80-160mcg/day |
| Fluticasone propionate | 600-1000mcg/day | 300-500mcg/day | 100-250mcg/day |
| Fluticasone furoate | 200mcg/day | 100mcg/day | NA |

**Table E2.** Biologic criteria and minimum requirements for each criteria used in the current study.

| **Criteria*** | **Treatment before biologic** | **Exacerbations** |
| --- | --- | --- |
| UK | High-dose ICS plus ≥1 other controller | ≥ 3 |
| Global 1 | High-dose ICS plus ≥1 other controller | ≥ 1 |
| Global 2 | High-dose ICS plus ≥1 other controller | ≥ 2 |
| RCT 1 | Medium-dose ICS plus ≥ 1 other controller | ≥ 1 |
| RCT 2 | Medium-dose ICS plus ≥ 1 other controller | ≥ 2 |

All criteria were considered met if at least 70% of expected total inhaled corticosteroid (ICS) dose were prescribed.

**Table E3.** Summary of RCT eligibility criteria and rationale for the RCT1 and RCT2 definitions used in this study.

| **Study** | **Asthma Treatment** | **Exacerbation** | **Biomarker** | **Lung function** |
| --- | --- | --- | --- | --- |
| Omalizumab | | | | |
| Soler ERJ 2001 | High ICS+/-LABA | Any including 0 | Positive SPT, total IgE 30-700, | FEV1 ≥40% to 80% |
| Busse 2001 | Med-high ICS | Any including 0 | Positive SPT, total IgE 30-700, | FEV1 ≥40% to 80% |
| Humbert 2004 (INNOVATE) | High ICS+LABA, +/-OCS | Any including 0 | Positive SPT, total IgE 30-700 |  |
| Hanania 2011 (EXTRA) | High ICS+LABA, +/-mOCS | Any including 0 | Positive SPT, total IgE 30-700, | FEV1 ≥40% to 80% |
| Mepolizumab | | | | |
| Pavord 2012 (DREAM) | High ICS+/-LABA+/-OCS, mOCS included | ≥2 | sputum eos ≥3%, feno ≥50, BEC ≥300 |  |
| Ortega 2014 (MENSA) | Severe asthma | ≥2 | BEC ≥150 at screening, ≥300 prev year | FEV1 <80% or ratio <80% |
| Bel 2014 (SIRIUS) | High ICS+controller on mOCS |  | BEC ≥150 at screening, ≥300 prev year |  |
| Reslizumab | | | | |
| Castro 2015 | Med-high ICS, mOCS included | ≥1 | BEC ≥400 |  |
| Bkermer 2016 | Med-high ICS, mOCS excluded | poor ACQ-7 | BEC ≥400 |  |
| Corren 2016 | Med-high ICS, mOCS excluded | "inadequately controlled" | None |  |
| Benralizumab | | | | |
| Bleecker 2016 (SIROCCO) | Med-high ICS+LABA +/-mOCS | ≥2 | None | FEV1<80% |
| Fitzgerald 2017 (CALIMA) | Med-high ICS+LABA +/-mOCS | ≥2 | None | FEV1<80% |
| Nair 2017 (ZONDA) | Med-high ICS+LABA + mOCS at least 6m |  | BEC ≥150 |  |
| Dupilumab | | | | |
| Wenzel 2016 | Med-high ICS+LABA |  | BEC ≥300 | FEV1 40-80% predicted at screening |
| Castro 2018 (LIBERTY ASTHMA QUEST) | Med-high ICS+LABA, LABA, LRTA | ≥1 | None | FEV1=<80% predicted at screening |
| Rabe 2018 (LIBERTY ASTHMA VENTURE) | High ICS+LABA/LAMA, **on mOCS** 6m |  | None | FEV1=<80% predicted at screening |
| Tezepelumab | | | | |
| Corren 2017 (PATHWAY) | Med-high ICS+LABA, mOCS included | ≥2 | None | FEV1 ≥40% to 80% at screening |
| Menzies-Gow 2021 (NAVIGATOR) | Med-high ICS+/- controller +/- mOCS | ≥2 | None | FEV1<80% at screening |
| Wechsler 2022 (SOURCE) | Med-high ICS+LABA+/-controller, on mOCS 6m included | ≥1 | None | FEV1<80% at screening |

| **Summary of criteria applied in RCTs above** | **Criteria used for this study in RCT1 and RCT2** |
| --- | --- |
| Asthma severity and therapies: most trials used medium–high dose ICS + ≥1 other controller. | Medium-dose ICS |
| Exacerbations: most trials used either ≥1 or ≥2 exacerbations in the prior year. | RCT1 set criteria at ≥1, RCT2 set criteria at ≥2 |
| Maintenance OCS: some studies required ≥6 months mOCS. | ≥12 OCS prescriptions |
| Biomarker criteria: most anti-IL-5 trials used blood eosinophils (BEC) ≥300/µL (often within the prior year). Reslizumab used >400/µL. Two mepolizumab and one benralizumab study used ≥150/µL at screening. Tezepelumab did not require biomarker thresholds. Omalizumab used SPT and IgE. | Multiple BEC thresholds: ≥150/µL, ≥300/µL and >400/µL |
| Lung function: most trials used FEV1 ≥40% to 80% at screening. | Not included |

**Definitions: ATS/ERS SA**, American Thoracic Society/European Respiratory Society–defined severe asthma; **BEC**, blood eosinophil count; **ICS**, inhaled corticosteroids; **LABA**, long-acting β₂-agonists; **LAMA**, long-acting muscarinic antagonists; **mOCS**, maintenance oral corticosteroids.

**Table E4.** Summary of global eligibility prevalence studies and rationale for the GLOBAL1 and GLOBAL2 definitions used in this study.

| **Study** | **Region** | **Asthma Treatment** | **Exacerbation** | **Biomarker** |
| --- | --- | --- | --- | --- |
| Omalizumab | | | | |
| Akenroye 2020 | USA | ICS+LABA/LAMA | ≥1 | USA FDA criteria |
| Albers 2018 | Global 6 countries | ATS/ERS SA | ≥1 or ≥2 12 months | Local licencing criteria |
| Buhl 2013 | Italy, Germany | High ICS+LABA | ≥2 in 12 months |  |
| Jeimy 2018 | Canada Ontario | ATS/ERS SA | None | Local licencing criteria |
| Kanniess 2021 | Europe 12 countries | ATS/ERS SA | None | Local licencing criteria |
| Lee 2019 | Korea | ATS/ERS SA | None | Korea FDA |
| Lee 2018 | Australia | ATS/ERS SA | None | licencing criteria |
| Marques Mello 2021 | Brazil | ATS/ERS SA | ≥1 | licencing criteria |
| Menzella 2020 | Italy | ATS/ERS SA | ≥1 in 12 months | licencing criteria |
| Anti-IL5s (including more than one anti IL-5 drug) | | | | |
| Akenroye 2020 | USA | ICS+LABA/LAMA | ≥1 in 3 months, ≥1 hospital in 12 months | FDA BEC criteria |
| Ilmarinen 2019 | Finland Seinajoki | Mod-high ICS+LABA | ≥2 in 12 months | BEC ≥300 or FeNO ≥50 |
| Kanniess 2021 | Europe 12 countries | ATS/ERS SA | European label criteria | European label criteria |
| Less 2018 | Australia | ATS/ERS SA |  | BEC ≥300 |
| Molfino 2020 | USA | Med-high ICS + LABA/LAMA | ≥2 in 12 months | BEC ≥150 in 12 months |
| Mepolizumab | | | | |
| Albers 2018 | Global 6 countries | ATS/ERS SA | ≥2 in 12 months | BEC ≥150 study visit or ≥300 past 12 months |
| Comberiati 2019 | USA | ATS/ERS SA | ≥2 in 12 months | BEC ≥150 or ≥300 |
| Jeimy 2018 | Canada Ontario | ATS/ERS SA | Inadequate control | BEC ≥150 or ≥300 in 12months |
| Marques Mello 2021 | Brazil | ATS/ERS SA | ≥2 in 12 months | BEC ≥150 |
| Menzella 2020 | Italy | ATS/ERS SA | ≥2 in 12 months | BEC ≥150 at screening or ≥300 in 12 months |
| Benralizumab | | | | |
| Jeimy 2018 | Canada Ontario | ATS/ERS SA | ≥2 in 12 months (or ≥1 on mOCS BEC>150) | BEC ≥300 in 12 months |
| Marques Mello 2021 | Brazil | ATS/ERS SA | ≥2 in 12 months | BEC ≥300 |
| Menzella 2020 | Italy | ATS/ERS SA | ≥2 in 12 months | BEC ≥300 in 12 months |
| Reslizumab | | | | |
| Albers 2018 | Global 6 countries | ATS/ERS SA | ≥1 in 12 months | BEC ≥400 at study visit |
| Jeimy 2018 | Canada Ontario | ATS/ERS SA | Inadequate control | BEC ≥400 |
| Marques Mello 2021 | Brazil | ATS/ERS SA | ≥1 | BEC ≥400 |
| Dupilumab | | | | |
| Akenroye 2020 | USA | ICS/LABA +/- LAMA 1y or mOCS 3m | ≥1 in 12 months | FDA criteria |
| Kanniess 2021 | Europe 12 countries | ATS/ERS SA | European label criteria | European label criteria |

| **Summary of criteria applied in published prevalence studies** | **Criteria used for this study in GLOBAL1 and GLOBAL2** |
| --- | --- |
| Asthma severity and therapies: most studies used ATS/ERS severe asthma defined as high-dose ICS + ≥1 other controller, or mOCS. | High-dose ICS + ≥1 other controller |
| Exacerbations: majority required ≥2 in the prior 12 months; a minority accepted ≥1. | GLOBAL1 set criteria at ≥1, GLOBAL2 set criteria at ≥2. |
| Biomarker criteria: BEC ≥300/µL within 12 months was the most common. Some accepted BEC ≥150/µL. Studies including reslizumab also used BEC ≥400/µL. No data on tezepelumab. Only one study (anti-IL5s) used FeNO. | Multiple BEC thresholds≥150/µL, ≥300/µL and >400/µL |

**Definitions: ATS/ERS SA**, American Thoracic Society/European Respiratory Society–defined severe asthma; **BEC**, blood eosinophil count; **ICS**, inhaled corticosteroids; **LABA**, long-acting β₂-agonists; **LAMA**, long-acting muscarinic antagonists; **mOCS**, maintenance oral corticosteroids.

**Table E5.** List and estimated prices for commonly used asthma biologics in the UK (with assumed NICE discounts)

Dosing and administration

- Tezepelumab: 210 mg subcutaneous (SC) every 4 weeks.
- Benralizumab: 30 mg SC every 4 weeks for the first 3 doses (weeks 0, 4, 8), then every 8 weeks thereafter.
- Mepolizumab: 100 mg SC every 4 weeks.
- Reslizumab: 3 mg/kg intravenous (IV) infusion every 4 weeks.

Tezepelumab costs were estimated on a per-dose basis. The first three doses are assumed to be administered in clinic, incurring a total administration cost of £171. Thereafter, all doses are assumed to be self-administered at home.

Annual costs for benralizumab, mepolizumab, and reslizumab were taken from the NICE review decision paper for TA479 (Ref: <https://www.nice.org.uk/guidance/ta479/evidence/review-decision-paper-pdf-9192962126>). For benralizumab and mepolizumab, costs are based on the TA671 committee meeting (slide 18), which assumes self-administration for all doses. For reslizumab, costs are also derived from TA671 (slide 18) and include intravenous administration, estimated at £1,064 in year 1 and £979 in year 2.

|  | **1 Dose** | **Year 1** | **Year 2** | **Total 5 Years** |
| --- | --- | --- | --- | --- |
| **Tezepelumab** | | | | |
| List price | 1265 | 15291 | 15180 | 76011 |
| 75% discount | 316 | 3823 | 3795 | 19003 |
| **Benralizumab** | | | | |
| List price | 1955 | 15753 | 12708 | 66585 |
| 75% discount | 489 | 3938 | 3177 | 16646 |
| **Mepolizumab** | | | | |
| List price | 840 | 11033 | 10920 | 54713 |
| 75% discount | 210 | 2758 | 2730 | 13678 |

**Table E6.** Estimated cost of exacerbation episodes

| **Event** | **Cost per episode** | **Source** |
| --- | --- | --- |
| GP consultation with prednisolone prescription 40mg for 5 days | £37 + £2 | King’s fund |
| ED visit by ambulance | £459 | King’s fund |
| Hospital admission | £3373 | NICE TA880 used this figure (2020/2021 National Cost Collection non-elective HRGs) |

**Table E7.** Annual costs of biologic treatment and post-treatment exacerbations over a 5-year horizon for all asthma patients in England meeting UK, Global and RCT criteria, starting from 20% biologic treatment penetration with 10% annual incremental increases, and corresponding annual cost-savings from avoided exacerbations.

**UK criteria (n=54,577)**

| **Year** | **Uptake Rate** | **Patients on Biologic** | **Cost for Biologics (£)** | **Exac Cost Not On Biologic (£)** | **Exac Cost On Biologic (£)** | **Exac Cost (£)** | **Net Annual Cost (£)** |
| --- | --- | --- | --- | --- | --- | --- | --- |
| 1 | 20.0% | 10,915 | £33,892,863 | £41,240,564 | £2,577,535 | £43,818,100 | £77,710,962 |
| 2 | 30.0% | 16,373 | £50,839,323 | £36,085,494 | £3,866,303 | £39,951,797 | £90,791,119 |
| 3 | 40.0% | 21,831 | £67,785,754 | £30,930,423 | £5,155,071 | £36,085,494 | £103,871,248 |
| 4 | 50.0% | 27,289 | £84,732,186 | £25,775,353 | £6,443,838 | £32,219,191 | £116,951,376 |
| 5 | 60.0% | 32,746 | £101,678,617 | £20,620,282 | £7,732,606 | £28,352,888 | £130,031,505 |
| **5-Year Total** | | | **£338,928,742** | **£154,652,116** | **£25,775,353** | **£180,427,469** | **£519,356,211** |

**Global 2 (n=90,892)**

| **Year** | **Uptake Rate** | **Patients on Biologic** | **Cost for Biologics (£)** | **Exac Cost Not On Biologic (£)** | **Exac Cost On Biologic (£)** | **Exac Cost (£)** | **Net Annual Cost (£)** |
| --- | --- | --- | --- | --- | --- | --- | --- |
| 1 | 20.0% | 18,178 | £56,444,841 | £68,681,631 | £4,292,602 | £72,974,233 | £129,419,074 |
| 2 | 30.0% | 27,268 | £84,667,290 | £60,096,427 | £6,438,903 | £66,535,330 | £151,202,620 |
| 3 | 40.0% | 36,357 | £112,889,711 | £51,511,223 | £8,585,204 | £60,096,427 | £172,986,138 |
| 4 | 50.0% | 45,446 | £141,112,131 | £42,926,019 | £10,731,505 | £53,657,524 | £194,769,655 |
| 5 | 60.0% | 54,535 | £169,334,551 | £34,340,815 | £12,877,806 | £47,218,621 | £216,553,173 |
| **5-Year Total** | | | **£564,448,524** | **£257,556,116** | **£42,926,019** | **£300,482,135** | **£864,930,659** |

**Global1 (n=160,005)**

| **Year** | **Uptake Rate** | **Patients on Biologic** | **Cost for Biologics (£)** | **Exac Cost Not On Biologic (£)** | **Exac Cost On Biologic (£)** | **Exac Cost (£)** | **Net Annual Cost (£)** |
| --- | --- | --- | --- | --- | --- | --- | --- |
| 1 | 20.0% | 32,001 | £99,364,705 | £120,906,178 | £7,556,636 | £128,462,814 | £227,827,519 |
| 2 | 30.0% | 48,002 | £149,047,086 | £105,792,906 | £11,334,954 | £117,127,860 | £266,174,946 |
| 3 | 40.0% | 64,002 | £198,729,439 | £90,679,634 | £15,113,272 | £105,792,906 | £304,522,345 |
| 4 | 50.0% | 80,003 | £248,411,791 | £75,566,361 | £18,891,590 | £94,457,952 | £342,869,743 |
| 5 | 60.0% | 96,003 | £298,094,144 | £60,453,089 | £22,669,908 | £83,122,998 | £381,217,141 |
| **5-Year Total** | | | **£993,647,165** | **£453,398,168** | **£75,566,361** | **£528,964,530** | **£1,522,611,695** |

**RCT2 (n=176,853)**

| **Year** | **Uptake Rate** | **Patients on Biologic** | **Cost for Biologics (£)** | **Exac Cost Not On Biologic (£)** | **Exac Cost On Biologic (£)** | **Exac Cost (£)** | **Net Annual Cost (£)** |
| --- | --- | --- | --- | --- | --- | --- | --- |
| 1 | 20.0% | 35,371 | £109,827,482 | £133,637,201 | £8,352,325 | £141,989,526 | £251,817,008 |
| 2 | 30.0% | 53,056 | £164,741,251 | £116,932,551 | £12,528,488 | £129,461,038 | £294,202,289 |
| 3 | 40.0% | 70,741 | £219,654,992 | £100,227,901 | £16,704,650 | £116,932,551 | £336,587,543 |
| 4 | 50.0% | 88,427 | £274,568,733 | £83,523,251 | £20,880,813 | £104,404,063 | £378,972,796 |
| 5 | 60.0% | 106,112 | £329,482,473 | £66,818,600 | £25,056,975 | £91,875,576 | £421,358,049 |
| **5-Year Total** | | | **£1,098,274,930** | **£501,139,503** | **£83,523,251** | **£584,662,754** | **£1,682,937,684** |

**RCT1 (n=328,144)**

| **Year** | **Uptake Rate** | **Patients on Biologic** | **Cost for Biologics (£)** | **Exac Cost Not On Biologic (£)** | **Exac Cost On Biologic (£)** | **Exac Cost (£)** | **Net Annual Cost (£)** |
| --- | --- | --- | --- | --- | --- | --- | --- |
| 1 | 20.0% | 65,629 | £203,780,705 | £247,958,732 | £15,497,421 | £263,456,153 | £467,236,858 |
| 2 | 30.0% | 98,443 | £305,671,087 | £216,963,891 | £23,246,131 | £240,210,022 | £545,881,109 |
| 3 | 40.0% | 131,258 | £407,561,440 | £185,969,049 | £30,994,842 | £216,963,891 | £624,525,330 |
| 4 | 50.0% | 164,072 | £509,451,792 | £154,974,208 | £38,743,552 | £193,717,760 | £703,169,552 |
| 5 | 60.0% | 196,886 | £611,342,145 | £123,979,366 | £46,492,262 | £170,471,628 | £781,813,773 |
| **5-Year Total** | | | **£2,037,807,169** | **£929,845,246** | **£154,974,208** | **£1,084,819,453** | **£3,122,626,622** |
